# A contemporary assessment of total neoadjuvant therapy (TNT) protocols for locally advanced rectal cancer: adoption and expert perspectives at German Cancer Society (DKG)-certified colorectal cancer centers

**DOI:** 10.1007/s00432-023-05139-6

**Published:** 2023-07-12

**Authors:** Melanie Langheinrich, Christoph Paasch, René Mantke, Klaus Weber, Stefan Benz, Stephan Kersting

**Affiliations:** 1 Department of Surgery, University Hospital Greifswald, 17475 Greifswald, Germany; 2 Department of Surgery, Brandenburg Medical School, University Hospital Brandenburg/Havel, 14770 Brandenburg, Germany; 3 Department of Surgery, University Hospital Erlangen, 91054 Erlangen, Germany; 4 Department of Surgery, Hospital Böblingen, 71032 Böblingen, Germany

**Keywords:** Rectal cancer, Locally advanced rectal cancer, Total neoadjuvant therapy, TNT, DKG-certified colorectal cancer centers

## Abstract

**Purpose:**

The treatment paradigm for locally advanced rectal cancer (LARC) is shifting toward the total neoadjuvant therapy (TNT) concept, which administered systemic chemotherapy in the neoadjuvant setting, either before or after chemoradiotherapy (CRT) or short-course radiotherapy (SCRT). First results have shown higher pathologic complete response (pCR) rates and a favorable impact on disease-free survival (DFS). Our study aimed to evaluate the current clinical practice and expert opinion regarding TNT for locally advanced rectal cancer across DKG (German Cancer Society)-certified colorectal cancer centers.

**Methods:**

A comprehensive online questionnaire, constituted of 14 TNT-focused queries targeting patients with locally advanced rectal cancer, was conducted among DKG-certified colorectal cancer centers registered within the database of the Addz (Arbeitsgemeinschaft Deutscher Darmzentren) between December 2022 and January 2023.

**Results:**

A significant majority (68%) indicated that they treated between 0 and 10 patients using a TNT protocol. Only a third (36%) of these centers participated in patient enrollment for a TNT study. Despite this, 84% of centers reported treating patients in a manner analogous to a TNT study, with the RAPIDO regimen being the most prevalent approach, employed by 60% of the respondents. The decision to adopt a TNT approach was primarily influenced by factors, such as the lower third of the rectum (93% of centers), cT4 stage (86% of centers), and a positive circumferential resection margin (80% of centers). Regarding concerns, 65% of the survey respondents expressed no reservations about the TNT concept, while 35% had concerns. In particular, there appears to be disagreement and uncertainty in regard to a clinical complete response and the “Watch and Wait” approach. While some centers adopt the watch-and-wait approach (42%), others only utilize it when extirpation is otherwise necessary (39%), and a portion still proceeds with surgery as initially planned (19%). The survey also addressed unmet needs, which were elaborated in the free-text responses. Overall, there was high interest in participating in planned observational studies.

**Conclusions:**

This study presents an overview of current clinical practice and unmet needs within DKG-certified German colorectal cancer centers. It is noteworthy that total neoadjuvant therapy (TNT) is predominantly performed outside of clinical trials. Moreover, across the centers, there is significant heterogeneity in handling clinical complete response and adopting the “watch and wait” approach. Further research is needed to establish standardization in the care of locally advanced rectal cancer.

**Supplementary Information:**

The online version contains supplementary material available at 10.1007/s00432-023-05139-6.

## Background

Globally, colorectal cancer (CRC) is one of the third most common cancers among both sexes, 30% of cases developing in the rectum. Over the past few decades, the approach to treating locally advanced rectal cancer (LARC) has remarkably evolved. For many years, the standard treatment for LARC consisted of neoadjuvant long-course chemoradiotherapy or short-course radiotherapy followed by surgery. During the last 20 years, this multimodal treatment has led to a significant reduction in local recurrence rates, reaching approximately 5% (Hofheinz 2021; Kreis et al. 2020; Phillips et al. 1984; Sauer et al. 2012). However, the rate of distant metastases remained constant, fluctuating around 20–30%, and thus, overall survival could only be slightly improved (Breugom et al. 2015).

Recently, new therapeutic strategies have emerged in an effort to further refine multimodal treatment and improve patient outcomes. Due to the absence of consistent data, international guidelines do not provide a uniform recommendation for adjuvant chemotherapy, and adherence to adjuvant chemotherapy has generally been low. To guarantee effective chemotherapy, particularly in terms of systemic control, preoperative chemotherapy has been intensified as part of the total neoadjuvant therapy (TNT) concept. At ASCO 2020, the first results from phase III studies were presented. Chemotherapy can be administered before or after R(Ch)T (induction or consolidation therapy). There are concepts with 5 × 5 Gy followed by FOLFOX/CAPOX (RAPIDO trial, consolidation chemotherapy) or others with mFOLFIRINOX followed by a capecitabine-based chemoradiotherapy (PRODIGE-23 trial, induction chemotherapy) (Bahadoer et al. 2021; Conroy et al. 2021). These studies have shown that TNT leads to a significant improvement in disease-free survival (DFS) as well as a significant improvement in the rate of pathological complete remissions (pCR). Moreover, the sequence of TNT, whether as consolidation versus induction chemotherapy, impacts the pCR rate (ACO-ARO-AIO-12) as well as organ preservation rates (OPRA) (Fokas et al. 2019; Garcia-Aguilar et al. 2022).

According to a consensus recommendation from the Association of Surgical Oncology (ACO), the Association of Radiooncology (ARO), and the Association of Internal Oncology (AIO), TNT is now the preferred new treatment option for patients with locally advanced rectal cancer. In line with this, the German Rectal Cancer Study Group (GRCSG) also advised that further data and follow-up are essential to confirm oncological safety and functional outcomes before integrating the “watch and wait” (W&W) approach into regular clinical practice for patients with a cCR.

The objective of this present appraisal is to examine the current state and expert viewpoints at colorectal cancer centers certified by the German Cancer Society (DKG), in anticipation of a forthcoming multicenter prospective observational trial.

## Methods

All DKG-certified colorectal cancer centers were invited to participate through the Working Group of German Colorectal Cancer Centers (ADDZ). The questionnaire was administered in December 2022 as an online survey and included 14 questions, attached as supplementary material. One single reminder was sent after a 4-week interval. To ensure data validity, responses were verified for plausibility, and submissions from identical IP addresses were only considered once.

## Results

**Fig. 1:**
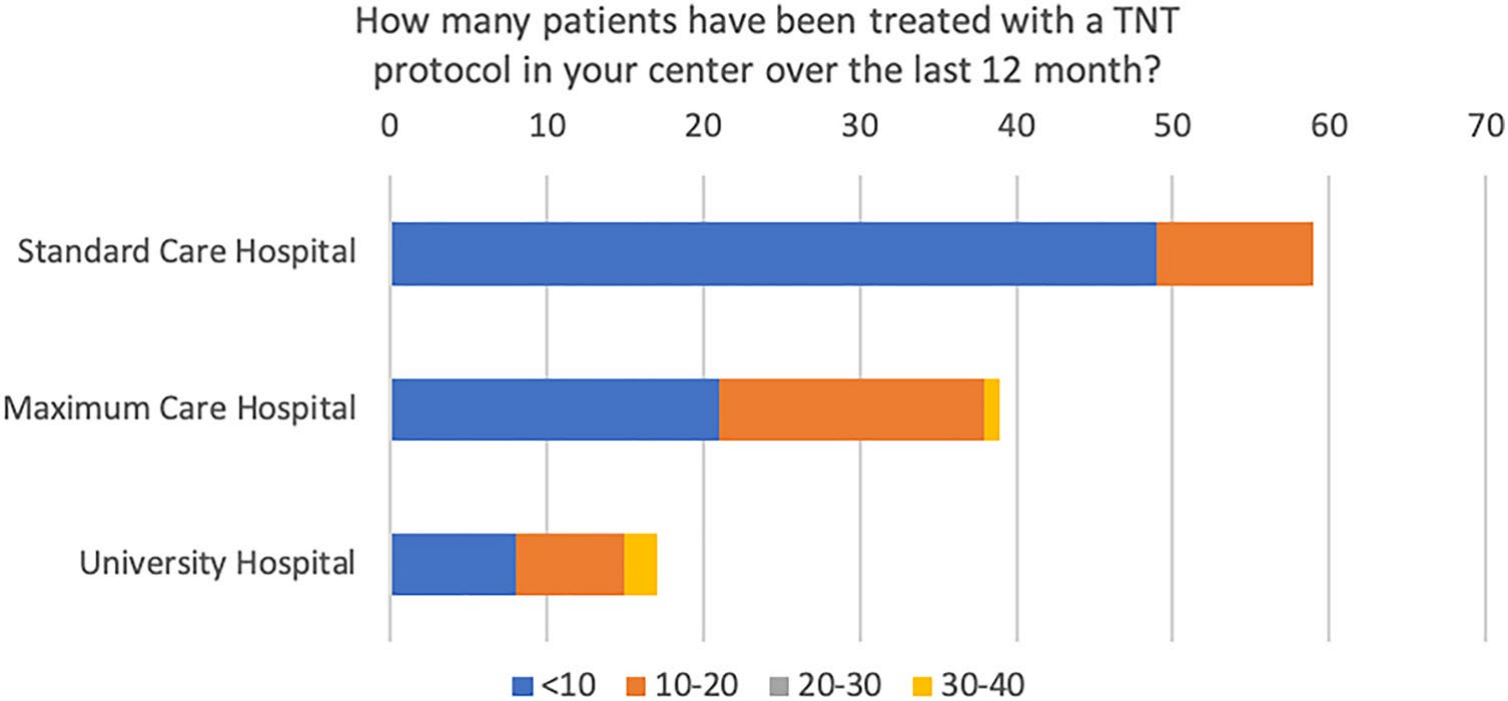
TNT case load based on cancer center location

**Fig. 2:**
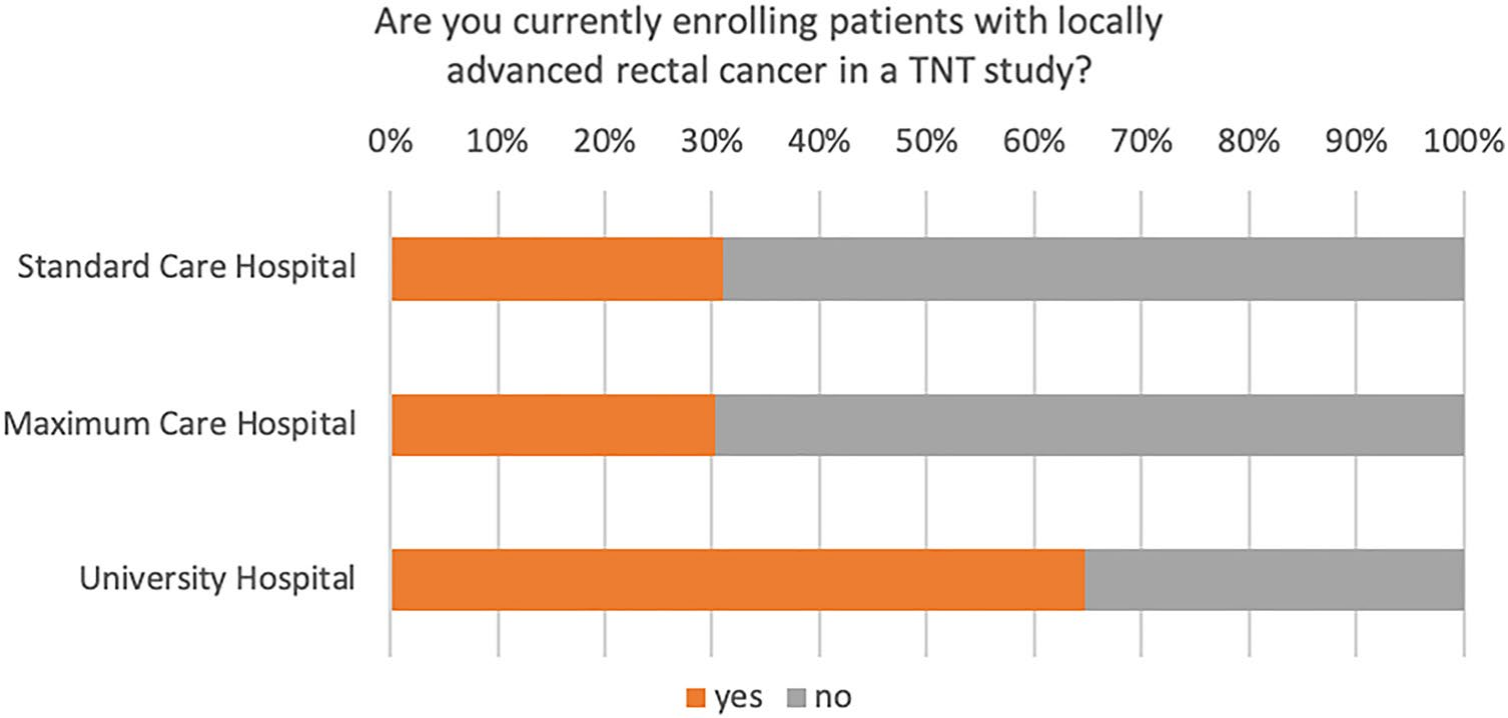
Proportion of respondents enrolling patients with locally advanced rectal cancer in TNT studies

**Fig. 3:**
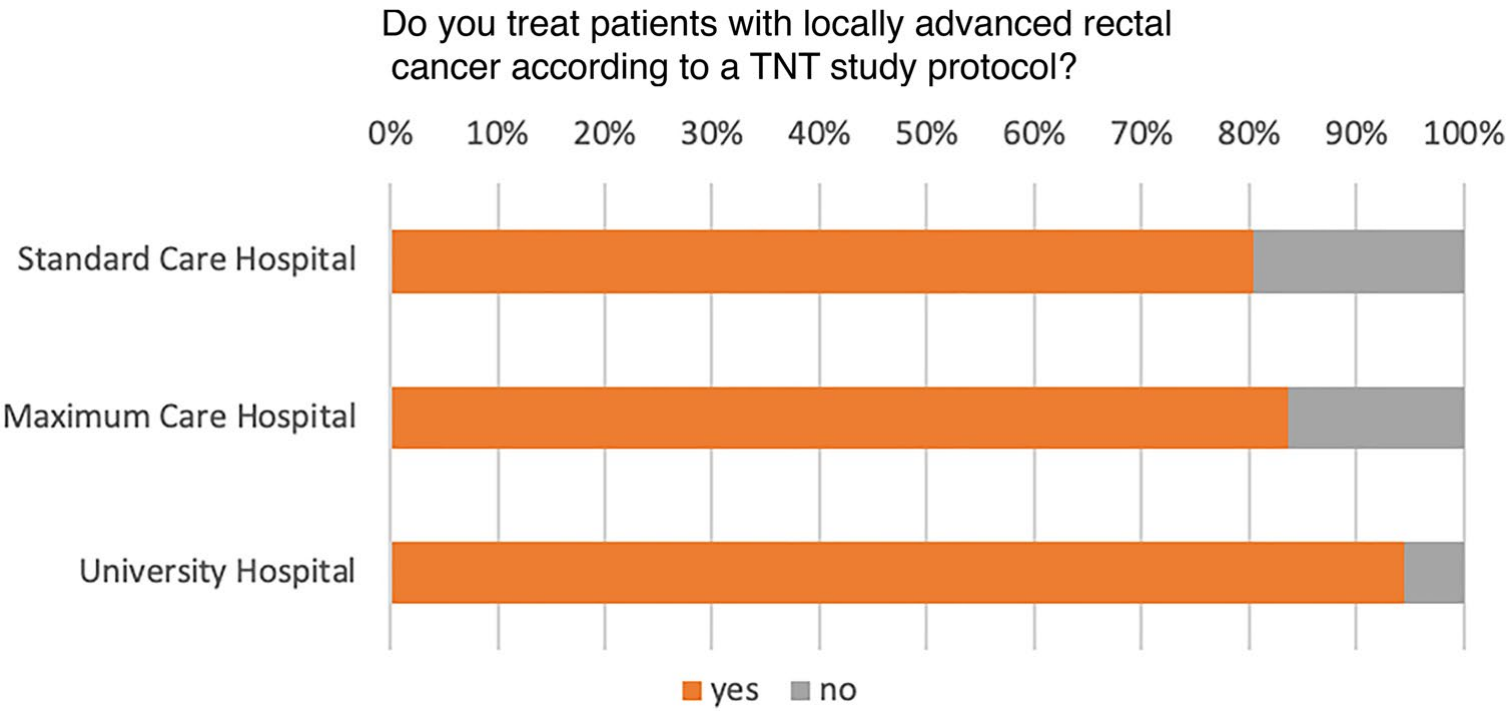
Percentage of respondents implementing TNT protocols for patients with locally advanced rectal cancer

**Fig. 4:**
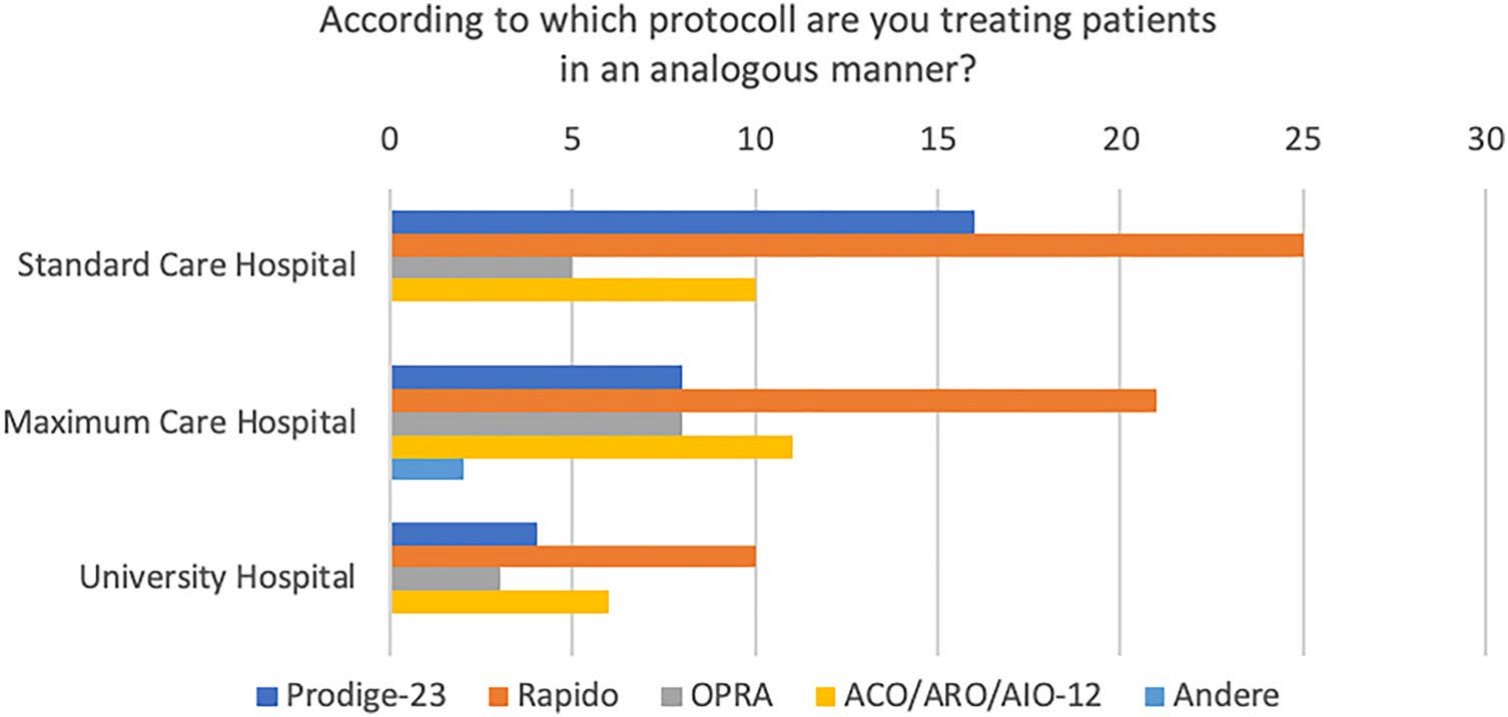
Breakdown of TNT protocols chosen by respondents

A total of 147 colorectal cancer centers contributed to the survey, consisting of 73 standard care hospitals, 54 maximum care hospitals, and 20 university hospitals. In 2021, these centers presented a variable spectrum of surgical primary cancer cases, with a majority (59%) reporting a caseload ranging from 20 to 30. Within this group, 68% of the centers indicated that they had treated between 0 and 10 patients using a TNT protocol. On the other hand, a substantial portion of the centers (28%) implemented the TNT protocol for a somewhat larger patient group, between 10 and 20 patients, as depicted in Fig. 1. Only a third (36%) of these centers enrolled patients in a TNT study (Fig. 2), with a vast majority (98%) participating in the ACO/ARO/AIO 18.1 study. Interestingly, even though only a limited number of patients were enrolled in TNT studies, 83% of the centers reported treating patients according to a TNT protocol (Fig. 3). Of these, 60% followed the RAPIDO regime, and 30% adhered to the PRODIGE-23 protocol (see Fig. 4).

**Fig. 5:**
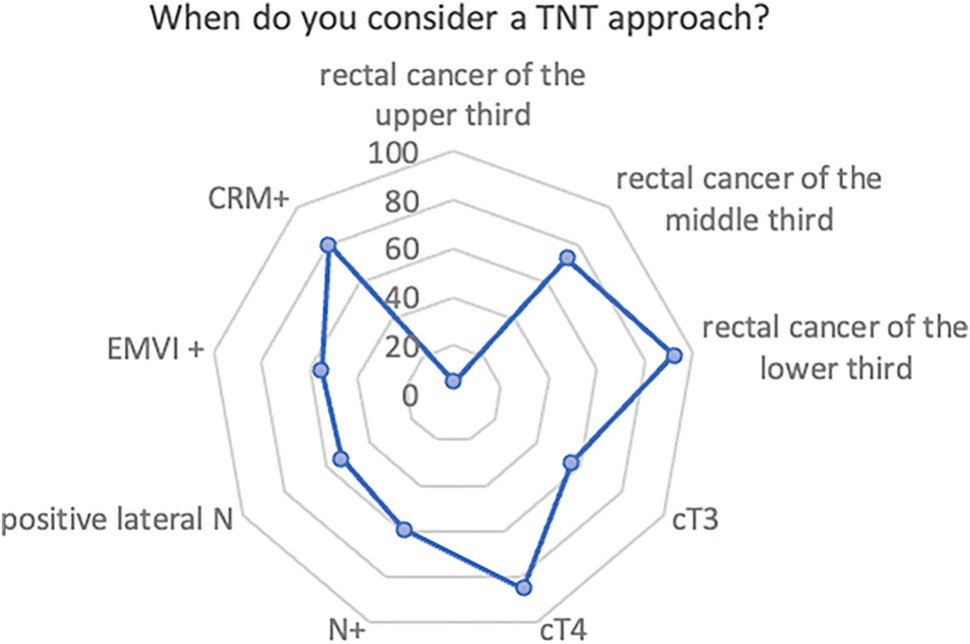
Importance of various characteristics in decision-making process: responses by percentage

The criteria influencing the decision to adopt a TNT approach are depicted in Fig. 5, with the lower third of the rectum (93%), cT4 stage (86%), and positive circumferential resection margin (80%) being the major factors.

**Fig. 6:**
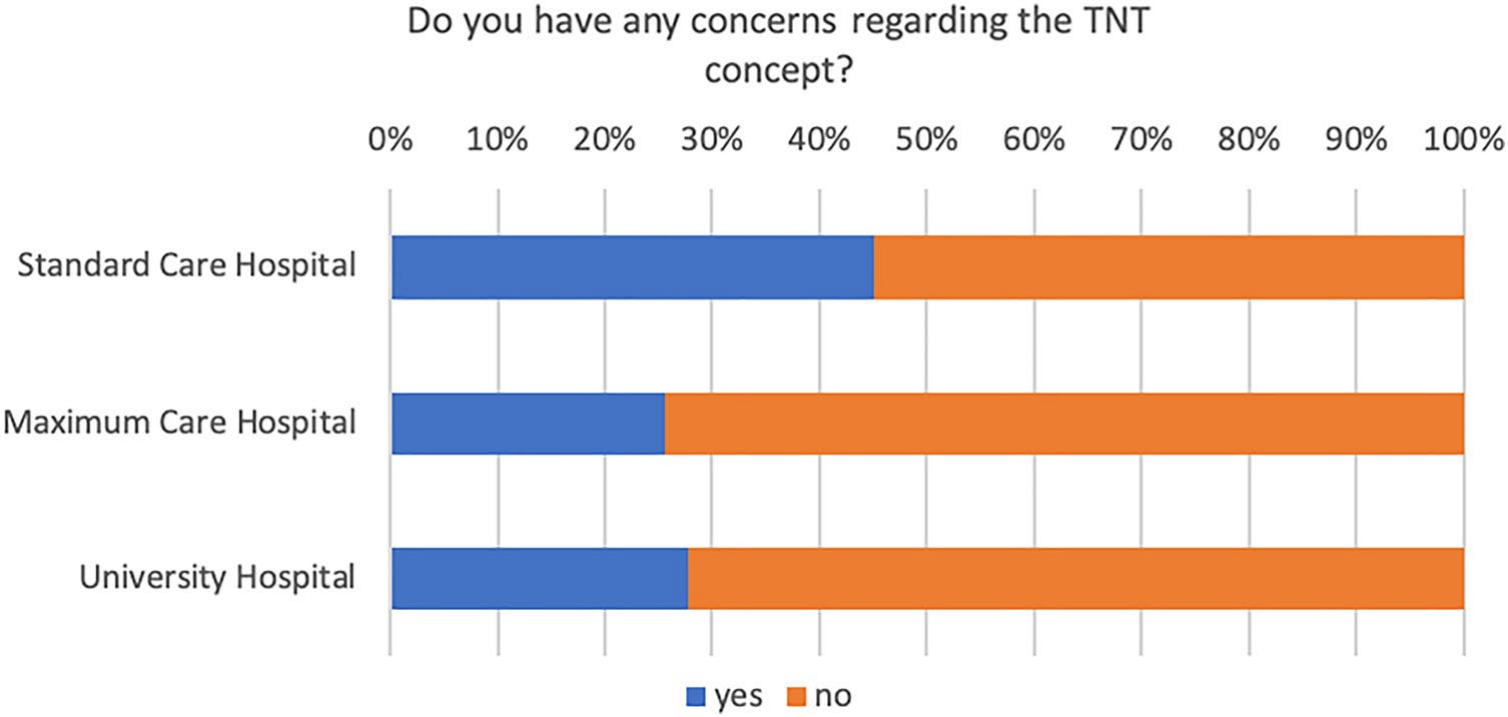
Respondent’s perspectives on TNT

**Fig. 7:**
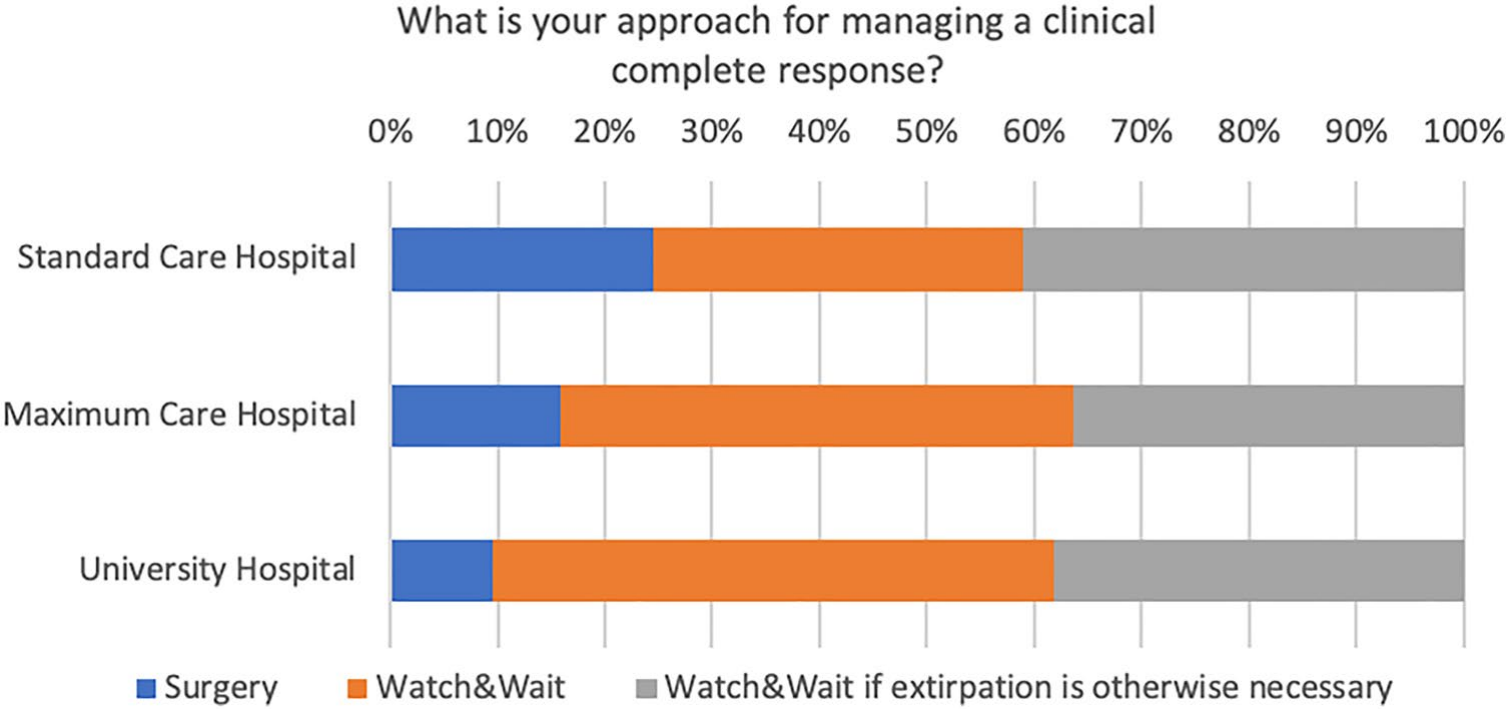
Strategies adopted by respondents for managing clinical complete response (cCR)

Among the survey respondents, a significant majority (65%), expressed no reservations about the TNT concept, while 35% of the respondents articulated concerns. Figure 6 shows the frequency of responses by location of the center. These concerns were specifically associated with potential toxicities, the evolution of a low anterior resection syndrome (LARS), and the time interval between radiation and surgery, which correlates with an increased incidence of pelvic fibrosis and edema. The free-text responses provided additional elaboration on these concerns. Notably, the “Watch and Wait” strategy, variable definitions of complete clinical remission (cCR) and response assessment, optimal timing for therapy response evaluation, the possibility of overstaging, and the subsequent risk of overtreatment were emphasized. In cases of clinical complete remission (cCR), 42% adopt a watch-and-wait approach, while 39% only use this approach when extirpation is otherwise necessary and 19% proceed with surgery (Fig. 7). A significant majority, 86%, expressed high interest in participating in a planned observational study.

## Discussion

Total neoadjuvant therapy is a novel approach for locally advanced rectal cancer. Emerging data from TNT studies demonstrate higher rates of pathological complete response (pCR), potential benefits in terms of distant metastasis control, and disease-free survival (DFS) (Donnelly et al. 2023; Sclafani et al. 2021; Goffredo et al. 2022). However, the optimal sequence of treatments remains a contentious subject, and to date, no proven survival benefit (OS) has been demonstrated.

The present survey is the first to capture the current state of care for the total neoadjuvant therapy concept in German colorectal cancer centers, accredited by DKG. Our findings reveal that TNT is already being implemented in clinical practice at these centers, despite not yet being fully incorporated into the existing guidelines. In the majority of centers (60%), regardless of their classification as standard care hospitals, maximum care hospitals, or university hospitals, the RAPIDO regimen is the preferred treatment protocol for TNT. Remarkably, TNT is mostly being performed outside of clinical trials in the participating centers.

The survey also addressed the ‘Watch and Wait’ approach in the context of handling patients who achieve clinical complete remission (cCR). The findings clearly demonstrated differing perspectives and a lack of consensus on this approach among the respondents. For instance, while 19% of the respondents would proceed with surgery as initially planned, approximately 39% considered ‘Watch and Wait’ as a feasible strategy if abdominoperineal resection (APR) is required. These variations in approaches to handling cCR, particularly the differing opinions on the use of the ‘Watch and Wait’ approach, highlight a significant level of uncertainty or disagreement in the field. This disparity in practice could signal a need for further research and the development of more definitive, consensus-driven guidelines or protocols for managing cCR in the context of locally advanced rectal cancer.

Moreover, the free-text responses clearly indicate the existence of several key issues that demand attention. These include the proper timing for reassessments during treatment, strategies for managing a clinical complete response, the development of precise clinical and radiological criteria for its accurate identification, potential risks and management of late toxicity associated with TNT, the need for additional biomarkers to guide therapeutic decisions, and the creation of a standardized approach for patient counseling and shared decision-making about the benefits and potential risks of the TNT strategy.

Given the complexity of these subjects, it becomes crucial to encourage a collective initiative or dialog that actively involves oncologists across various specialties, such as radiological, medical, radiation, and surgical fields. This concerted effort, coupled with comprehensive research, serves a dual purpose: it aims to address these substantial issues and challenges while also enhancing our understanding of LARC toward the design of individualized patient therapies, tailoring the treatment approach to each patient´s characteristics, unique circumstances, and needs.

Despite some limitations, our study holds considerable significance. While participation was confined to DKG-certified colorectal cancer centers, it confers a certain level of standardization and quality assurance to the practices surveyed. The robust response rate, ranging from standard care hospitals to maximum care and university hospitals, underscores the relevance and representativeness of our results within this professional community. Our results not only offer substantial and valuable insights into current practice patterns but also expose the unmet needs voiced by experts in the field, thus addressing open questions and knowledge gaps regarding the application of total neoadjuvant therapy (TNT) for locally advanced rectal cancer. By illuminating present practices, our study lays a robust foundation for future research, driving the development and ultimately leading to improved outcomes out quality of life for patients with locally advanced rectal cancer.

In conclusion, our findings reveal that total neoadjuvant therapy (TNT) is being widely adopted in clinical practice at DKG-certified German colorectal cancer centers, even outside of clinical trials. This highlights the pressing need for detailed attention to key areas that require further clarification and refinement. Such fields encompass the optimal timing for reassessment of cCR, the establishment of precise criteria for managing clinical complete response, and delineating guidelines for the Watch-and-Wait approach. By addressing these crucial aspects, we can advance our understanding and implementation of TNT in the management of locally advanced rectal cancer, ultimately improving patient outcomes.

## Supplementary Information

Below is the link to the electronic supplementary material.

Supplementary file1 (DOCX 15 KB)

## Data Availability

The datasets generated during and/or analyzed during the current study are available from the corresponding author on reasonable request.
