# Supplementary material for: A contemporary assessment of total neoadjuvant therapy (TNT) protocols for locally advanced rectal cancer: adoption and expert perspectives at German Cancer Society (DKG)-certified colorectal cancer centers
Source: J Cancer Res Clin Oncol. 2023 Jul 12;149(13):12591–6. doi: 10.1007/s00432-023-05139-6 (PMC10465655; doi:10.1007/s00432-023-05139-6)
Supplement: Supplementary file 1 — Supplementary file1 (DOCX 15 KB) [file 432_2023_5139_MOESM1_ESM.docx]

**Supplementary file**

**Question 1**

***Please specify the location of your colorectal cancer center***

- Standard Care Hospital
- Maximum Care Hospital
- University Hospital

**Question 2**

***Please specify your qualification: certified Colorectal Surgeon?***

- YES
- NO (please forward the survey link to the colorectal surgeon of your center)

**Question 3**

***Please specify the total number of primary cases of the rectum in 2021***

- <20
- 20-30
- 30-40
- 40-50
- 50-60
- 60-70
- 70-80
- >80

**Question 4**

***How many patients with locally advanced rectal cancer have you treated in your center following a TNT protocol in the past 12 months?***

- <10
- 10-20
- 20-30
- 30-40
- 40-50
- >50

**Question 5**

***Are you currently enrolling patients with locally advanced rectal cancer in a study for total neoadjuvant therapy?***

- YES
- NO

**Question 6**

***Into which total neoadjuvant therapy (TNT) study are you enrolling patients?*** *(You can select multiple options)*

- ACO/ARO/AIO-18.1
- Other: free text option

**Question 7**

***Do you treat patients with locally advanced rectal cancer according to a total neoadjuvant therapy (TNT) study protocol?***

- YES
- NO

**Question 8**

***According to which regimen do you treat the patients following a study protocol****? (You can select multiple options)*

- Prodige-23
- Rapido
- OPRA
- ACO/ARO/AIO-12
- Other: free text option

**Question 9**

***When do you consider a TNT approach?*** *(You can select multiple options)*

- Rectal cancer of the upper third
- Rectal cancer of the middle third
- Rectal cancer of the lower third
- cT3
- cT4
- Lymph node positive (N+)
- Extramural vascular invasion (EMVI+)
- Positive circumferential resection margin/ mesorectal fascia invasion (CRM/MRF +)
- At least two of the mentioned factors must be positive
- More than two of the mentioned factors must be positive
- Other: free text option

**Question 10**

***Do you have any concerns regarding the TNT concept?***

- YES
- NO

**Question 11**

***What are your concerns?*** *(You can select multiple options)*

- Time interval between radiation and surgery
- Grade 3/4 toxicities
- Postoperative complication rate
- Pelvic fibrosis, edema
- TME quality
- Quality of life (e.g. LARS)
- Deviation from the established own standard
- Other: free text option

**Question 12**

***What is your approach for managing a clinical complete response?*** *(You can select multiple options)*

- Surgery
- Watch & Wait if extirpation is otherwise necessary
- Watch & Wait

**Question 13**

***Do you have a structured watch-and-wait follow-up program at your colorectal cancer center?***

- YES
- NO

**Question 14**

***Are you interested in participating in a planned observational study on total neoadjuvant therapy (TNT)?***

- YES
- NO
